# COVID-19: Integrating genomic and epidemiological data to inform public health interventions and policy in Tasmania, Australia

**DOI:** 10.5365/wpsar.2021.12.4.878

**Published:** 2021-12-22

**Authors:** Nicola Stephens, Michelle McPherson, Louise Cooley, Rob Vanhaeften, Mathilda Wilmot, Courtney Lane, Michelle Harlock, Kerryn Lodo, Natasha Castree, Torsten Seemann, Michelle Sait, Susan Ballard, Kristy Horan, Mark Veitch, Fay Johnston, Norelle Sherry, Ben Howden

**Affiliations:** a Tasmanian School of Medicine, University of Tasmania, Tasmania, Australia.; b Public Health Services, Tasmanian Department of Health, Tasmania, Australia.; c Menzies Institute for Medical Research, University of Tasmania, Tasmania, Australia.; d Microbiological Diagnostic Unit Public Health Laboratory, Department of Microbiology & Immunology, University of Melbourne at the Doherty Institute, Victoria, Australia.; e Tasmanian Health Services, Tasmanian Department of Health, Tasmania, Australia.

## Abstract

**Objective:**

We undertook an integrated analysis of genomic and epidemiological data to investigate a large health-care-associated outbreak of coronavirus disease 2019 (COVID-19) and to better understand the epidemiology of COVID-19 cases in Tasmania, Australia.

**Methods:**

Epidemiological data collected on COVID-19 cases notified in Tasmania between 2 March and 15 May 2020, and positive samples of severe acute respiratory syndrome coronavirus 2 (SARS-CoV-2) or RNA extracted from the samples were included. Sequencing was conducted by tiled amplicon polymerase chain reaction with ARTIC v1 or v3 primers and Illumina sequencing. Consensus sequences were generated, sequences were aligned to a reference sequence and phylogenetic analysis was performed. Genomic clusters were determined and integrated with epidemiological data to provide additional information.

**Results:**

All 231 COVID-19 cases notified in Tasmania during the study period and 266 SARS-CoV-2-positive samples, representing 217/231 (94%) notified cases, were included; 184/217 (84%) were clustered, 21/217 (10%) were unique and 12/217 (6%) could not be sequenced. Genomics confirmed the presence of seven clusters already identified through epidemiological links, clarified transmission networks in which the epidemiology had been unclear and identified one cluster that had not previously been recognized.

**Discussion:**

Genomic analysis provided useful additional information on COVID-19 in Tasmania, including evidence of a large health-care-associated outbreak linked to an overseas cruise, the probable source of infection in cases with no previously identified epidemiological link and confirmation that there was no identified community transmission from other imported cases. Genomic insights are an important component of the response to COVID-19, and continuing genomic surveillance is warranted.

Genomic sequencing for characterization of severe acute respiratory syndrome coronavirus 2 (SARS-CoV-2) was developed early during the coronavirus disease 2019 (COVID-19) pandemic. (*1*, *2*) Since then, genomics has been used internationally to understand the dynamics of viral transmission (*3*) and the genetic evolution of the virus. (*4*-*6*) Locally, genomic analysis has been used to analyse transmission routes, assign likely origins of infection, link outbreak cases and inform public health interventions and policies. (*7*-*11*)

Integrated analysis of genomic and epidemiological data provides additional benefits for public health investigations (*12*-*14*) and has been used during the COVID-19 pandemic. (*9*, *14*-*16*) Genomic sequencing of SARS-CoV-2 polymerase chain reaction (PCR)-positive diagnostic samples combined with epidemiological data has been shown to be beneficial in investigating health-care-associated infections, (*9*, *17*) monitoring community transmission, (*8*-*10*) informing public health responses (*9*, *10*, *18*) and understanding the pathology of the disease. (*9*, *10*, *18*)

In Australia, integration of genomic sequencing into the response to COVID-19 has allowed clusters and outbreaks to be identified and transmission chains to be rapidly detected. (*9*) Genomic data enhance national surveillance data by clarifying the source of infection in outbreak settings and in cases with no known source of infection, by characterizing clusters of disease transmission (*5*) and by providing evidence of the introduction of lineages into Australia and any changes in cases acquired locally and overseas. (*19*)

Tasmania, an island state of Australia with a population of approximately 540 000, had one of Australia’s first documented health-care-associated outbreaks of COVID-19. The first case of COVID-19 in Tasmania was notified on 2 March 2020. By 2 April 2020, a total of 80 cases had been notified, the distribution approximating the geographical distribution of the population throughout the state. Epidemiological investigations indicated that most infections had been acquired overseas (68/80, 85%), with a small number acquired locally after exposure to a known case (4/80, 5%) and 8 (10%) cases under investigation at the time (Internal reports, Department of Health Tasmania, 2020). On 3 April 2020, two cases were notified in health-care workers (HCWs) in a hospital in north-western Tasmania, and a third was notified the following day. These three cases signalled the beginning of a large outbreak that occurred among three health-care facilities and resulted in 138 cases. (*20*, *21*) At the time, the outbreak of COVID-19 was the largest to have occurred in a health-care facility in Australia, and public health investigations were critical to both control the outbreak and inform future public health actions.

To provide further evidence for the public health investigation and management of the outbreak in north-western Tasmania and to better understand the epidemiology of all COVID-19 cases in the state, the Tasmanian Department of Health in collaboration with the Microbiological Diagnostic Unit Public Health Laboratory (MDU) undertook an integrated analysis of genomic and epidemiological data for COVID-19 cases in Tasmania. This paper describes the findings.

## Methods

COVID-19 cases notified to the Tasmanian Department of Health between 2 March and 15 May 2020 were included in the analysis. PCR-positive samples for SARS-CoV-2, or extracted RNA if such samples were not available, were referred to the MDU with any epidemiological data that had been collected and were stored in the Tasmanian Government’s COVID-19 database. Epidemiological clusters were defined as two or more COVID-19 cases that were linked by person, place and/or time, cases linked to an international cruise or cases linked to an interstate cluster.

The epidemiological data were analysed with STATA v14. They comprised demographics; onset date; whether the case resided in an aged-care facility or was a health or aged-care worker and, if so, whether they had worked in the 24 hours and/or 14 days before onset; whether the case was linked to a cluster and, if so, the outbreak code; whether they had travelled overseas or interstate and the countries or jurisdictions visited; whether they had had contact with a known case; and place of acquisition (if known) or whether no source was identified.

Sequencing and phylogenetic analyses were conducted as described by Seemann et al. Briefly, RNA extracted from SARS-CoV-2 reverse transcription PCR-positive samples underwent tiled amplicon PCR with ARTIC (version 1 or 3) primers. Sequencing libraries were prepared from amplicons with NexteraXT and sequenced on Illumina NextSeq. Reads were aligned against a SARS-CoV-2 reference genome (MN9008947.3 Wuhan Hu-1), and consensus sequences were generated. Quality control for consensus sequences included requiring 80% of the genome to be recovered, 25 single nucleotide polymorphisms from the reference genome and £300 ambiguous or missing bases. Sequences with 65–80% genome recovery were assessed for potential inclusion in the phylogenetic analysis. A maximum likelihood algorithm was used for phylogenetic reconstruction. Genomic clusters were determined with ClusterPicker and curated with the cleaned epidemiological data. Each confirmed case was assigned a genomic cluster identifier which was uploaded onto the Tasmanian COVID-19 database. Further analysis was conducted with STATA v14 to compare epidemiological clusters with the identified genomic clusters, unique cases and those that could not be sequenced.

## Results

### Epidemiological clusters

Twelve epidemiological clusters were identified in Tasmania before the genomic analysis. One was a cluster seeded from a returned international traveller (EC01), six were linked to separate overseas cruises (EC02–EC06, EC12), one was a case linked to an interstate cluster (EC07) and four were part of the north-western outbreak – the main outbreak of 129 cases and smaller linked clusters at an aged-care facility, within the community and at an additional hospital (EC08–EC11) (**Table 1**).

**Table 1:** Tasmanian COVID-19 epidemiological and genomic clusters, 2 March–15 May 2020

Epidemiological cluster ID	Number of epidemiologically linked cases	Epidemiological links	Genomic cluster
EC01	3	Index case acquired overseas; transmission on local cruise	C
EC02	22	Overseas cruise A	A.1 and A.2
EC03	15 (14 on cruise plus one secondary case)	Overseas cruise B	B
EC04	1	Overseas cruise C	Not clustered
EC05	1	Overseas cruise D	Sequencing failed
EC06	9	Overseas cruise E	D
EC07	1	Local case linked to interstate cluster	G (one of the three cases in this genomic cluster)
EC08	129	North-western outbreak	A.1
EC09	1	North-western outbreak cluster 1; aged-care facility (index case in EC08)	A.2
EC10	6	North-western outbreak cluster 2; community cluster (index case in EC08)	A.2
EC11	2	North-western outbreak cluster 3; additional hospital (index case in EC08)	A.2
EC12	1	Overseas cruise F	Not clustered

Note: Not all cases linked to the epidemiological clusters were submitted for genomic analysis; therefore, the numbers of cases per epidemiological cluster do not always add up to the number by genomic cluster.

### Genomic clusters

The 266 SARS-CoV-2-positive samples were referred to the MDU, representing 217 of the 231 cases (94% of all cases) notified during the study period. Fourteen samples were not referred because of insufficient sample volume or very high cycle threshold (correlated with low levels of virus in the sample). Of the 217, 184 were part of a genomic cluster, 21 were unique (singletons) and 12 could not be sequenced (i.e. did not meet the sequencing quality control criteria).

**Figure 1:**
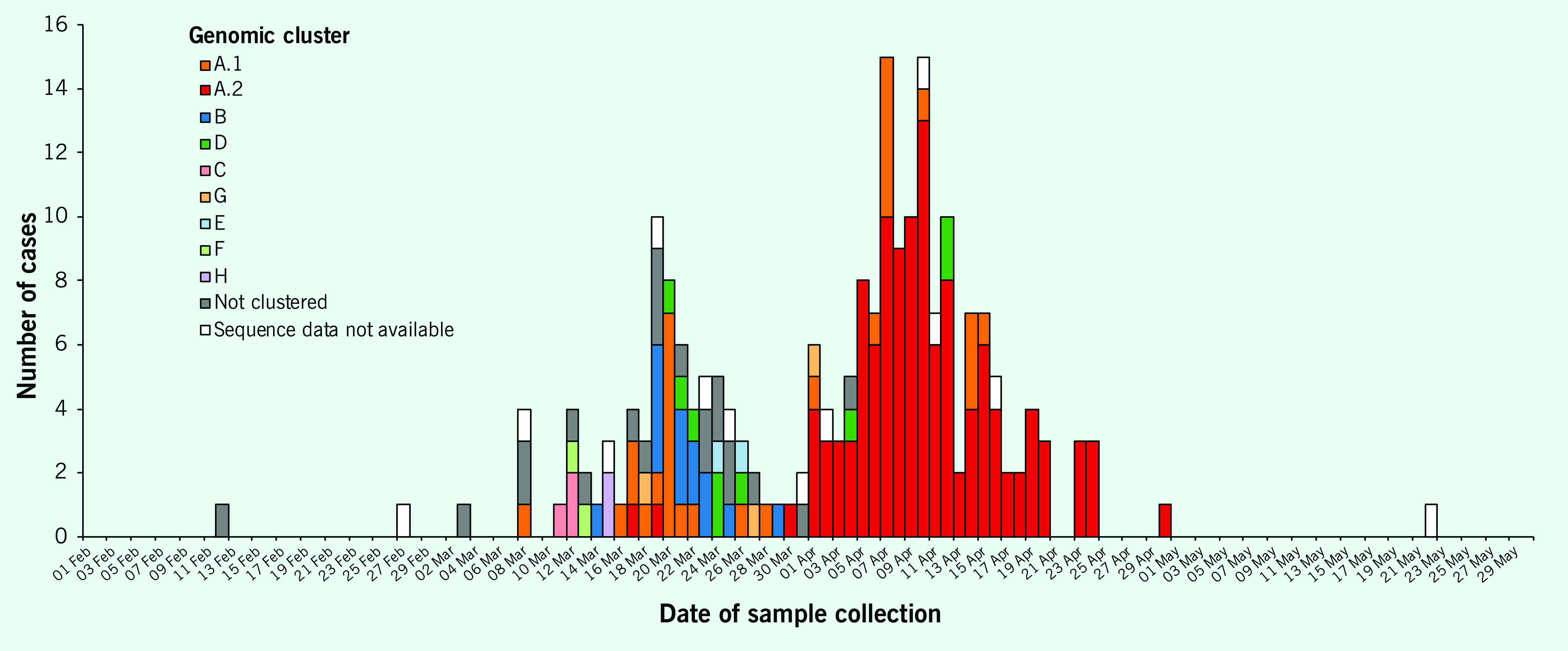
Epidemic curve of Tasmanian COVID-19 cases by genomic cluster

**Figure 2:**
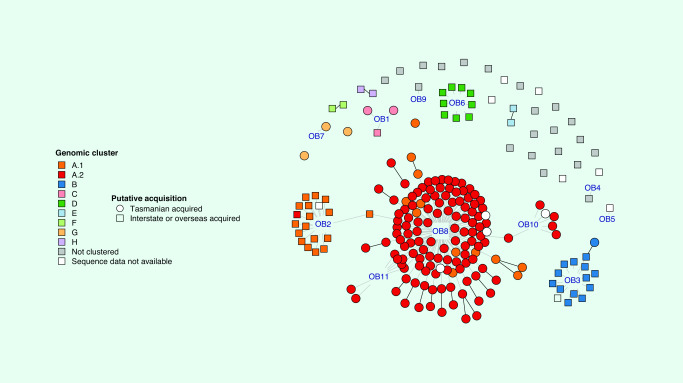
Tasmanian COVID-19 cases by epidemiological link, genomic cluster and putative source of acquisition, 2 March–15 May 2020

Eight genomic clusters were identified, clusters A–G (including two subclusters, A.1 and A.2), ranging in size from 2 to 149 cases (**Fig. 1**); all but one genomic cluster corresponded to epidemiological clusters or known travel partners (**Table 1**; **Fig. 2**).

[**insert** Figure 1]

### Genomic cluster A

The largest genomic cluster, cluster A, corresponded to cases from overseas cruise A (EC02) and the large north-western outbreak (EC08-EC11), confirming that the north-western outbreak was seeded from infections originally acquired on overseas cruise A. Two travellers on this cruise were admitted to hospital A in north-western Tasmania and were in genomic cluster A – one in each of the subgroups A.1 and A.2. The ship had travelled from Sydney to New Zealand with approximately 2700 passengers, of whom approximately 900 subsequently developed COVID-19. (*22*) This genomic cluster had two subgroups (A.1 and A.2 below) with dates of onset of 8 and 17 March, respectively (**Fig. 1**). Clusters A.1 and A.2 were very closely related, separated by one cluster-defining single nucleotide polymorphism.

### Genomic cluster A.1

Genomic cluster A.1 comprised 29 cases, including 17 returned overseas cruise A passengers (one of whom was admitted to hospital A and was thought to have been one of the index cases of the north-western outbreak), five HCWs from hospital A, six of their household contacts and a case not linked epidemiologically to the north-western outbreak. These corresponded to cases in EC02 and EC08.

Five cases in this cluster, all returned overseas cruise A passengers, were hospitalized (four at a hospital in southern Tasmania and one at hospital A), of whom two were admitted to an intensive care unit and two died. Three of the HCWs from hospital A reported having worked while symptomatic. The number of cases in cluster A.1 was highest in March, and cases continued to be detected until mid-April.

The unlinked case was a HCW from another hospital in north-western Tasmania, with no identifiable source of infection, despite extensive public health investigations. All HCWs who had worked at the hospital during their period of acquisition had done so before overseas cruise A docked in Sydney. The infection was thought to have been acquired during unidentified contact with a returned overseas cruise A passenger or a secondary case in the days before symptom onset. This case was not linked epidemiologically to any subsequent case.

### Genomic cluster A.2

Genomic cluster A.2 comprised 120 cases and consisted of another overseas cruise A passenger who was also admitted to hospital A and 119 cases associated with the north-western outbreak. This subcluster comprised 72 staff members, 23 patients and 24 of their contacts (linked to hospital cases but who were not admitted to the hospital) and the one overseas cruise A case, corresponding to one case from EC02 and cases from the other north-western outbreak clusters (EC08–EC11).

Of the 72 staff members, 57 worked at hospital A, six at a co-located private hospital and two at the neighbouring hospital; seven staff worked at more than one of these facilities. Five cases were part of a community cluster linked to hospital A (EC10), two were part of a cluster at the neighbouring hospital (EC11) and one from an aged-care facility was linked to a case at hospital A (EC09). Cluster A.2 was first detected in mid-March, with the outbreak peaking in the second week of April.

Almost one quarter of the cases (*n* = 28; 23%) were hospitalized, although 19 were infected as inpatients at the hospital, and one was admitted to an intensive care unit. There were 10 deaths: the returned cruise passenger and nine inpatients. Most of the cases in this subcluster (*n* = 95; 79%) reported having had contact with a confirmed case, and 72 had been identified as contacts before infection. The first three notified cases were in HCWs who had had no direct contact with a case. Twelve cases associated with the outbreak, including 10 HCWs, were already experiencing symptoms of COVID-19 by the time the first two hospital-acquired cases were notified to the Tasmanian Department of Health.

### Genomic clusters B–H

The remaining genomic clusters (B–H) ranged in size from 2 to 17 people, and, aside from cluster G, all had identified epidemiological links to specific sources, such as other cruise ships or travelling companions who had recently returned from interstate or overseas (**Tables 1** and **2**).

**Table 2:** Characteristics of COVID-19 genomic clusters, Tasmania, 2020

Genomic cluster ID (number of cases)	Onset date range (duration in days)	No. asymptomatic	No. in hospital	No. of COVID-19 deaths	No. of health-care workers^a^	Contact with COVID-19 case in 14 days before symptom onset	Identified as contact before infection	Place of acquisition
A.1 (*n* = 29)	8 March– 14 April (38)	1	5 (all cruise), 2 in ICU	2 (cruise)	6	27 (5 HS, 6 non-hospital, 17 other)	27 (4 HS, 6 non-hospital, 17 other)	17 overseas 12 Tasmania
A.2 (*n* = 120)	17 March– 24 April (39)	6	28 (6 HS, 20 patients)	10 (10 patients)	72	95 (56 HS, 16 patients, 23 other)	72 (41 HS, 10 patients, 20 non-hospital, 1 cruise)	1 overseas 119 Tasmania
B (*n* = 14)	14 March– 25 March (12)	1	0	0	3	14	14	13 overseas 1 Tasmania
C (*n* = 3)	11 March– 12 March (2)	0	2	0	0	3	2	1 overseas 2 Tasmania
D (*n* = 9)	20 March– 4 April (26)	0	1	0	1	9	9	9 overseas
E (*n* = 2)	24 March– 26 March (3)	0	1 ICU	0	0	1	1	2 Australia
F (*n* = 2)	12 March– 13 March (2)	0	0	0	0	0	0	2 overseas
G (*n* = 3)	18 March– 1 April (15)	0	0	0	0	1	1	3 Tasmania
H (*n* = 2)	15 March (1)	0	0	0	0	0	0	2 overseas
Non-clustered cases (*n* = 21)	12 February– 4 April (NA)	0	2	0	2	6	2	2 cruise 18 overseas 1 Australia^b^
N/S (*n* = 12)	27 February– 16 April (NA)	1	3 (2 NW outbreak)	1 (NW outbreak)	2	5	5 (2 cruise, 2 NW outbreak, 1 community cluster)	3 cruise 5 overseas 4 Australia^b^

N/S: sequencing not successful; HS: hospital staff; ICU: intensive care unit; NW: north-western

^a^ Indicates whether the case is a HCW, not where their infection was acquired.

^b^ Australia other than Tasmania

### Genomic clusters B and D

These two genomic clusters were associated with two separate overseas cruises, comprising 14 and nine cases, respectively, and corresponded to EC03 and EC06. All but one case in cluster B acquired COVID-19 while on the cruise. The additional case in cluster B was a contact of a case from the cruise.

### Genomic cluster C

This genomic cluster, comprising the cases from EC01, was associated with a group that travelled on a yacht tour of the east coast of Tasmania. The index case had just returned from an overseas trip to Canada, where they probably acquired COVID-19 infection. Two further cases were infected during the overnight tour.

### Genomic cluster E

Cluster E comprised two co-travellers within Australia who were linked epidemiologically but not defined as an epidemiological cluster. The onset of the two cases occurred within two days; one case was hospitalized.

### Genomic clusters F and H

Genomic clusters F and H also comprised two people each, who were co-travellers who had acquired their infection overseas. These two clusters were also linked epidemiologically but not defined as an epidemiological cluster. One couple had travelled to the USA and the other to Germany and the United Arab Emirates. None of these cases was hospitalized.

### Genomic cluster G

Genomic cluster G contained three cases not epidemiologically linked before the genomic analysis. One case was epidemiologically linked to two travellers from Queensland while infectious and corresponded to EC07, while the source of infection was not identified for the other two cases. Two of the cases were employed in jobs that required close contact with the public (taxi driver and tour bus driver), while the other was a tourist. All three were in the southern Tasmania area at the same time as the Queensland cases, although no clear epidemiological link was found between two of the cases and the Queensland travellers. Sequencing results uploaded to Australia’s platform for real-time analysis of integrated pathogen genomic data for public health, AusTrakka, (*23*) have since confirmed that the cluster G cases were closely related to interstate samples from Queensland, Victoria, New South Wales and South Australia.

### Non-clustered cases

There were 21 cases with unique genomic sequences and onset dates between 12 February and 4 April 2020. The group included four of the initial cases notified in Tasmania (**Fig. 1**). All were travel-related cases: two cases had travelled on different cruise ships (one each from EC04 and EC12), 18 had travelled internationally and one had travelled to Victoria. The cases had visited 15 different countries, and nine had travelled to several countries (**Table 2**, **Fig. 2**). Six cases (29%) reported having had contact with confirmed COVID-19 cases: two were household contacts and four were travel contacts. All the cases were symptomatic, and two were hospitalized; there were no deaths.

[**insert** Figure 2]

There were two cases in the group that had travelled together, each initially nominated as a contact of the other. They had travelled to Europe (Austria, England and Italy); they had onset of infection days apart but had unrelated genomic sequences.

### Cases that could not be sequenced

Samples from 12 cases could not be sequenced: seven were in the epidemiological clusters, and the remaining five had travelled overseas; none reported known contact with a confirmed COVID-19 case (**Fig. 2**). Those in known clusters included three from separate cruises (one each from EC02, EC03 and EC05) and four from the north-western outbreak (two patients and one staff member from EC08 and one that was part of the community cluster EC10). The onset dates ranged from 27 February to 16 April (**Fig. 1**).

## Discussion

We used genomic sequencing to add further evidence to the epidemiological data collected on COVID-19 cases in Tasmania, Australia. We were able to illustrate transmission routes within the state, from when the first case was notified through to when Tasmania effectively eliminated the virus. We found 31 groups of SARS-CoV-2 genomic sequences in 217 cases notified in Tasmania (eight genomic clusters, one split into two subclusters and 23 singletons unrelated to other cases by genomics), reflecting the broad travel histories associated with the cases.

The most valuable information provided by this study was that a large health-care-associated outbreak in north-western Tasmania was seeded from overseas cruise A, as initially hypothesized in the case series review. (*20*) Two separate transmission pathways were identified from overseas cruise A passengers admitted to hospital to HCWs, which then spread to two other hospital campuses, to close contacts of the HCW cases and to a limited extent into the community. This genomic cluster continued from early March to late April and ended after initiation of control measures, including hospital closure, cleaning and disinfection, a 14-day regional lockdown, quarantining of contacts and their households and screening of hospital staff before they returned to work.

Three cases linked by genomic analysis were not previously epidemiologically linked, suggesting limited community transmission relatively early in the outbreak in Tasmania (18 March to 1 April), when most other cases were in returned international travellers. These three cases were linked geographically and temporally and had exposures related to travel or tourists. More recent sequencing has shown that these cases are linked to interstate samples, demonstrating the importance and utility of sequence-sharing between jurisdictions for public health. Similarly, a previously unrelated case was linked to the first subcluster of the overseas cruise A/health-care-associated outbreak. After intensive review of the data, community transmission is also considered to be the most likely source of infection in this case.

Genomic analysis added value by quantifying the effectiveness of Tasmania’s public health interventions. Aside from the transmission described above, genomic analysis found no evidence of community transmission in Tasmania by the other 113 cases in returned travellers, highlighting the success of quarantine, contact-tracing and testing procedures in the state.

Integration of genomic sequence data with epidemiological data improves understanding of SARS-CoV-2 transmission patterns and outbreak dynamics. (*24*) Routine inclusion of genomic data into public health surveillance can inform interventions and monitor their success, (*9*) indicate the likely source of infection in outbreaks or in cases with no known source and highlight patterns of transmission in populations. (*25*) The analyses were conducted retrospectively in Victoria; however, Tasmania has since developed genomic capacity locally, which will improve the timeliness of future outbreak investigations. Genomics can also play an important part in monitoring the evolution of SARS-CoV-2 over time and changes in its pathogenicity, immunogenicity or transmissibility. (*25*, *26*) Genomic surveillance will also be critical in monitoring selective pressure from vaccines as they are rolled out. (*26*, *27*)

A major strength of our study was our ability to combine genomic sequence with epidemiological data for 94% of the Tasmanian COVID-19 cases. A high rate of genomic sequencing was achieved because genomic surveillance programmes were already in place for other priority public health pathogens, with strong partnerships and capabilities among key organizations, providing the necessary infrastructure, governance and referral arrangements and laboratory expertise for rapid development and scaling-up of genomic surveillance for COVID-19. These working relationships will be crucial to the success of continuous genomic surveillance, use of genomics in the prevention and control of future  SARS-CoV-2 outbreaks (*10*) and the development of local genomics capacity.
